# *In vivo* Induction of Functional Inhibitory IgG Antibodies by a Hypoallergenic Bet v 1 Variant

**DOI:** 10.3389/fimmu.2020.02118

**Published:** 2020-09-03

**Authors:** Lorenz Aglas, Athanasios Bethanis, Paulina Chrusciel, Frank Stolz, Melanie Gruen, Ulla-Marjut Jaakkola, Laurian Jongejan, Emrah Yatkin, Ronald Van Ree

**Affiliations:** ^1^Department of Biosciences, University of Salzburg, Salzburg, Austria; ^2^Central Animal Laboratory, University of Turku, Turku, Finland; ^3^Biomay AG, Vienna Competence Center, Vienna, Austria; ^4^Amsterdam University Medical Centers, Amsterdam, Netherlands

**Keywords:** hypoallergen, Bet v 1, birch pollen, AIT, IgG, blocking, allergy, PFAS

## Abstract

Allergic sensitization to the major allergen Bet v 1 represents the dominating factor inducing a vast variety of allergic symptoms in birch pollen allergic patients worldwide, including the pollen food allergy syndrome. In order to overcome the huge socio-economic burden associated with allergic diseases, allergen-specific immunotherapy (AIT) as a curative strategy to manage the disease was introduced. Still, many hurdles related to this treatment exist making AIT not the patients’ first choice. To improve the current situation, the development of hypoallergen-based drug products has raised attention in the last decade. Herein, we investigated the efficacy of the novel AIT candidate BM4, a hypoallergenic variant of Bet v 1, to induce treatment-relevant cross-reactive Bet v 1-specific IgG antibodies in two different mammals, Wistar rats and New Zealand White rabbits. We further analyzed the cross-reactivity of BM4-induced Wistar rat antibodies with the birch pollen-associated food allergens Mal d 1 and Cor a 1, and the functional capability of the induced antibodies to act as IgE-blocking IgG antibodies. Enzyme-linked immunosorbent assay (ELISA) was used to determine the titers of rat IgG1, IgG2a, IgG2b, and IgE, as well as rabbit IgG and IgE antibodies. To address the functional relevance of the induced IgG antibodies, the capacity of rat sera to suppress binding of human IgE to Bet v 1 was investigated by using an inhibition ELISA and an IgE-facilitated allergen-binding inhibition assay. We found that the treatment with BM4 induced elevated Bet v 1-specific IgG antibody titers in both mammalian species. In Wistar rats, high BM4-specific IgG1, IgG2a, and IgG2b titers (10^4^ to 10^6^) were induced, which cross-reacted with wild-type Bet v 1, and the homologous allergens Mal d 1 and Cor a 1. Rat allergen-specific IgG antibodies sustained upon treatment discontinuation. Sera of rats immunized with BM4 were able to significantly suppress binding of human IgE to the wild-type allergens and CD23-mediated human IgE-facilitated Bet v 1 binding on B cells. By contrast, treatment-induced IgE antibody levels were low or undetectable. In summary, BM4 induced a robust IgG immune response that efficiently blocked human IgE-binding to wild-type allergens, underscoring its potential therapeutic value in AIT.

## Introduction

Birch pollen (*Betula verrucosa*) represents the major elicitor of tree pollen-associated allergic symptoms in Europe, with sensitization patterns ranging from 54 up to 92% among patients allergic to tree pollen (1–3). The peculiarity that the IgE-mediated pathology is dominantly triggered by the recognition of the allergen Bet v 1 – reactivity rates around 95% – prompted researchers world-wide to develop therapeutic approaches targeting this protein (4). Besides seasonal occurring symptoms, such as rhinoconjunctivitis and allergic asthma, birch pollen allergic patients frequently report oral and food allergy symptoms, creating a complex disease endotype. These symptoms are elicited by plant food sources including fruits, vegetables and nuts, and occur due to the recognition of similar structural motifs shared between food allergens and the primary sensitizing pollen allergen. The estimated prevalence of symptoms triggered by such food allergens ranges between 50 and 90% among birch pollen allergic patients (5–9). This clinical manifestation is described as the pollen food allergy syndrome (PFAS) (5). Apples and hazelnuts belong to the most common allergenic sources amongst plant foods eliciting birch pollen-associated PFAS symptoms (8, 10, 11). Here, the culprit allergens responsible for triggering PFAS are the Bet v 1-homologous pathogenesis-related protein-10 (PR-10) class proteins Mal d 1 (apple, *Malus domestica*) and Cor a 1 (hazelnut, *Corylus avellana*). Although the adverse health effects caused by birch pollen are efficiently treatable by allergen-specific immunotherapy (AIT), the concomitant treatment of PFAS symptoms induced by Bet v 1-associated food sources remains debatable, highlighting strengths, and possibilities for therapeutic improvements (12–16). Other disadvantages of AIT are long treatment duration, the risk of side-effects and, in case of subcutaneously applied AIT, repeated injections into the patient’s skin, resulting in a poor treatment compliance (17).

In order to address these challenges in AIT, we have developed a hypoallergenic variant of Bet v 1, termed BM4, possessing beneficial characteristics including decreased allergenicity (reduced binding by Bet v 1-specific IgE) and ameliorated immunogenicity (enhanced T cell-activating capacity and proliferation) (18–20). BM4 was genetically engineered through the substitution of five amino acids in the wild-type Bet v 1 sequence by corresponding residues in the Mal d 1 sequence, resulting in a general collapse of the otherwise globular PR-10 fold. In turn, these structural changes altered conformation-dependent IgE epitopes (18). The EU-funded project “BM4SIT – Innovations for Allergy” aimed to evaluate the efficacy of BM4 as an AIT vaccine candidate in a first-in-men clinical trial. Drug toxicity studies, preceding human clinical trials, are used to investigate the safety profile of vaccine candidates in various mammalian species in order to estimate patients’ tolerability, and, thus, are a mandatory step toward drug approval. In the course of the BM4 toxicity studies, conducted in Wistar rats and New Zealand White (NZW) rabbits, we undertook a detailed profiling of the BM4-induced humoral immune response in a naïve setting. It is well established that the reduction of symptoms in AIT, and consequently the improvement of the patient’s quality of life, are mainly accomplished by the induction of immunotolerance, which is maintained by allergen-specific blocking IgG4 and partially by IgG1 antibodies able to neutralize IgE-mediated allergen binding (21–24). Therefore, the objective of the present study was to perform a pre-clinical evaluation of the Bet v 1-specific IgG as well as IgE immune response induced by immunizations with BM4 in both Wistar rats and NZW rabbits. We further analyzed the functionality of the induced IgG antibodies regarding their capability to inhibit human IgE-facilitated binding of Bet v 1-IgE complexes to B cells, a confirmed biomarker for AIT efficacy (25, 26). Additionally, we investigated the cross-reactivity of the serum antibodies toward the Bet v 1-associated food allergens Mal d 1 and Cor a 1.

Herein, we show that immunizations of Wistar rats and NZW rabbits with BM4 resulted in high levels of IgG antibodies cross-reactive to wild-type Bet v 1. In Wistar rats, functional Mal d 1- and Cor a 1-cross-reactive IgG antibodies were induced, however, to a lesser extent compared to the Bet v 1. In rats receiving repeated immunizations with BM4, sustained IgG antibody titers remained even upon treatment discontinuation. In addition, BM4-induced IgG antibodies displayed a functional inhibitory activity toward the binding of human IgE to the wild-type pollen allergen (Bet v 1) and the associated food allergens (Mal d 1 and Cor a 1).

## Materials and Methods

### Recombinant Proteins

BM4 (designated BM41 by the manufacturer Biomay AG, Vienna, Austria) was produced recombinantly and endotoxin-free under GMP conditions based on the described protocol (18). Both BM4 and placebo were formulated using aluminum hydroxide (Alu-Gel-S, Serva, Heidelberg, Germany). Expression, purification, physicochemical characterization, and determination of endotoxin contamination (<0.3 ng/mL) of recombinant Bet v 1.0101, Mal d 1.0108, and Cor a 1.0401 (called Bet v 1, Mal d 1, and Cor a 1 in the following) were performed as previously described (27, 28). A representative SDS-PAGE image showing the different recombinant proteins can be found in Supplementary Figure S1.

### Analysis of Secondary Structural Elements

The structural composition of the recombinant proteins was determined using circular dichroism (CD) and Fourier transform infrared (FTIR) spectroscopy. The CD spectra were recorded at 20°C between 190 and 260 nm using a JASCO J-815 spectropolarimeter (Jasco, Tokyo, Japan). All proteins were diluted in a 10 mM potassium phosphate buffer to a final concentration of 0.1 mg/ml. For the structural analysis using FTIR, the spectra in the range of the amide 1 and amide 2 peaks (1500–1700 cm^–1^) were recorded at a constant temperature (25°C) for each protein at concentrations of 1.5–2.0 mg/ml using an AquaSpec transmission cell adapted to a Tensor II FTIR system (Bruker Optics Inc., Billerica, MA, United States). The data were analyzed using the OPUS spectroscopy software 6.0 (Bruker Optics Inc., Billerica, MA, United States). The second derivative of the amide 1 spectra was calculated after vector-normalization (25 smoothing points) using the Savitzky–Golay algorithm.

### Animal Immunization Model(s)

Both species, NZW rabbits (Lidköpings kaninfarm, Lidköping, Sweden) and Wistar rats (RjHAN:WI, Janvier Labs, France), received a single subcutaneous (s.c.) injection (hereinafter “single immunization model”) of either 320 μg of BM4, formulated as 320 μg BM4/2 mg aluminum hydroxide/0.9% NaCl/ml, or the respective amount of adjuvant without antigen, termed “placebo” in the following (*n* = 5 male and *n* = 5 female per group). Since in human allergen-specific subcutaneous immunotherapy (SCIT) AIT vaccine is administered via the s.c. route of administration, BM4 was administered likewise. Treated animals were daily monitored in a specific pathogen-free (SPF) environment on a frequent basis for 14 days post-injection for clinical signs of test item effects, such as changes in skin and fur, respiration, eyes and mucous membranes, circulation and behavior patterns, and then sacrificed. Blood samples were collected pre- and 14 days post-injection and stored at −20°C until analysis. Sera of the single immunization model were only used for an initial screening of BM4-specific and Bet v 1-cross-reactive antibodies, whereas a further functional characterization of these sera was not pursued.

According to the OECD Guidelines for the testing of chemicals no 420, the rat is the preferred species. Therefore, the repeated toxicity study analyzing potential toxic effects of the hypoallergen was only conducted in Wistar rats (Charles River, Sulzfeld, Germany). Injections containing either 160, 80, 40, or 20 μg of BM4 (formulated as xx μg BM4/1 mg alum/0.9% NaCl/500 μl) were administered s.c. on a bi-weekly schedule over a period of 6 months (12 injections/animal in total); termed “repeated immunization model” in the following. This model was conducted in order to provide a immunization schedule relatable to a human AIT protocol. The animals of the main group (*n* = 10 per gender per treatment group) were sacrificed 1 week after the last injection and those in the recovery group (5 per gender per treatment group) 6 weeks after the last injection. The dose level used in the study was based on the doses employed in human AIT protocols.

All animal studies were conducted in compliance with the “OECD Principles of Good Laboratory Practice” (ENV/MC/CHEM(98)17) and the standard operating procedures (SOPs) of UTUCAL. All procedures and protocols were approved by the National Animal Experiment Board of Finland in accordance with the EU Directive 2010/63/EU on the protection of animals used for scientific purposes under the license numbers ESAVI/7217/04.10.03/2012, and ESAVI/8528/04.10.07/2015. UTUCAL animal facilities operate in compliance with the OECD Principles of Good Laboratory Practice (GLP) and have Animal Welfare Assurance by the Office of Laboratory Animal Welfare (OLAW), PHS, NIH. Assurance identification number is A5040-01.

### Enzyme-Linked Immunosorbent Assay

Endpoint titer of BM4-, Bet v 1-, Mal d 1-, or Cor a 1-specific IgE, IgG1, IgG2a, and IgG2b levels in rat sera were determined by enzyme-linked immunosorbent assay (ELISA). Either BM4, Bet v 1, Mal d 1, or Cor a 1 were coated in a concentration of 2 μg/ml diluted in 1× phosphate-buffered saline on Nunc MaxiSorp^®^ flat-bottom 96 well plates (Thermo Fisher Scientific, United States). Serial dilutions of rat sera were incubated with the respective antigen overnight at 4°C. Horseradish peroxidase (HRP)-conjugated mouse anti-rat IgG1, IgG2a, IgG2b (clones G1 7E7, 2a 8F4, and 2B 10A8, respectively, purchased from SouthernBiotech, Birmingham, United Kingdom), and IgE (clone MARE-1, Thermo Fisher Scientific, Rockford, IL, United States) antibodies diluted 1:5000 were used as detection antibodies. The SureBlue TMB (3,3′,5,5′-Tetramethylbenzidine) Microwell peroxidase substrate (KPL, Gaithersburg, MD, United States) was used for detection. Since NZW rabbits only possess a single IgG subclass, the BM4- and Bet v 1-specific rabbit IgG ELISAs were performed using a HRP-conjugated goat anti-rabbit IgG antibody, Fc Fragment (Jackson ImmunoResearch Inc., Suffolk, United Kingdom) for detection (29). Plates were measured at a wavelength of 450 nm using an ELISA Reader Infinite 200 PRO (Tecan, Switzerland). The limit of quantification (LOQ), defined as the sum of the mean plus the 10-fold standard deviation of the detection antibody controls, was used as cut-off for endpoint titer determination. The final antibody titers were calculated by plotting the experimental absorbance values against the dilution of a serum as previously described (30). For the determination of rabbit total IgE, an ELISA kit from BlueGene Biotech (Putuo District, Shanghai, China) was used according to the manufacturer’s instructions.

### Inhibition ELISA

For the inhibition ELISA, 2 μg/ml of either Bet v 1, Mal d 1, or Cor a 1 were coated on Nunc MaxiSorp^®^ flat-bottom 96 half-area well plates (Thermo Fisher Scientific, United States). After blocking with 0.5% BSA, the immobilized antigen was incubated with rat sera in dilutions of either 1:32 for Bet v 1 or 1:2 for Mal d 1, and Cor a 1 for 2h at room temperature. To exclude any potential interference of IgE antibodies, the rat sera were heat-inactivated at 56°C for 1h prior to the experimental procedure. After the inhibition step, the samples were incubated with different human reference serum pools per antigen (derived from birch allergic patients suffering from PFAS caused by apples and/or hazelnuts), diluted 1:2 for Bet v 1 and Mal d 1, and 1:8 for Cor a 1 (depending on the presence of specific IgE in the serum pools), overnight at 4°C. An alkaline phosphatase-conjugated mouse anti-human IgE antibody (clone B3102E8, SouthernBiotech, Birmingham, AL, United States), diluted 1:1000, and the alkaline phosphatase substrate p-nitrophenyl phosphate (Sigma-Aldrich, St. Luis, MI, United States) were used for detection. The absorbance was recorded at 405 nm after 3h of incubation at room temperature. The data were normalized to the uninhibited control (100%). The percentage of inhibitory activity of each individual serum was calculated by inverting the normalized absorbance value (100 minus normalized absorbance value) after background subtraction (detection antibody control).

### Inhibition IgE-Facilitated Allergen Binding (Inhibition FAB) Assay

We performed inhibition FAB assays to determine the capacity of BM4-induced rat antibodies to prevent the formation of Bet v 1-IgE complexes using a defined human indicator serum pool obtained from six individual birch pollen allergic patients. The indicator serum pool contained high levels of Bet v 1-specific IgE antibodies, as determined by ImmunoCAP assays (31). For this purpose, we used an adapted version of the protocol published by Shamji et al. (32). In short, IgE of rat sera was inactivated by heat treatment at 56°C for 1h prior to the experimental procedure. For the inhibition FAB assay, sera of animals receiving repeated immunizations of either 0 (placebo), 20, or 40 μg BM4 were used (main group: *n* = 20, recovery group: *n* = 10). A total of 15 μl of each rat serum was incubated with 10 ng/ml Bet v 1 for 1h at 37°C, followed by the addition of 1 μl of the human serum pool and further incubation for 1h at 37°C. Subsequently, 1 × 10^5^ Epstein–Barr virus (EBV)-transformed B cells expressing CD23, the low IgE-binding receptor (also called FcεRII), were added to the antibody allergen reaction mix. The cells were stained using a PE-labeled anti-human CD23 (BD Biosciences, San Jose, CA, United States) and a FITC-labeled anti-human IgE (KPL/medac GmbH, Wedel, Germany) in the dilutions 1:40 and 1:100, respectively. The experimental conditions, including antigen concentration, amount of human reference serum and the dilution of the FITC-labeled anti-human IgE antibody, were defined prior to the experiment (Supplementary Figure S3). To avoid false-negative results by dead cells, SYTOX^TM^ Red Dead Cell Stain (Thermo Fisher Scientific, Waltham, MA, United States) in a dilution 1:100 was used for live/dead (L/D) discrimination. As positive control, the uninhibited human serum pool (no rat serum, only Bet v 1 and human serum) was used for IgE-Bet v 1 complex formation. The cells were analyzed using a Cytoflex S (Beckman Coulter, Brea, CA, United States) and FlowJo v 10 (FlowJo, LLC, Ashland, OR, United States). Doublet discrimination was performed by gating the side scatter area (SSC-A) versus the SSC height (SSC-H). Only living EBV-transformed B cells were considered for the data analysis. The detailed gating strategy is shown in Supplementary Figure S4. The data were normalized to the mean of the uninhibited control (100%). The percentage of inhibitory activity of the sera was calculated by inverting the percentage obtained for the IgE-Bet v 1 complex formation (100 minus complex formation value) after baseline subtraction (mean of untreated cells). Compensation was performed and isotype as well as single stained controls were included in the experimental set-up. Ethical approval for using human serum of allergic patients was obtained by the Dutch ethical committee (number: NL65758.018.18).

### Statistical Analysis

**FIGURE 1:**
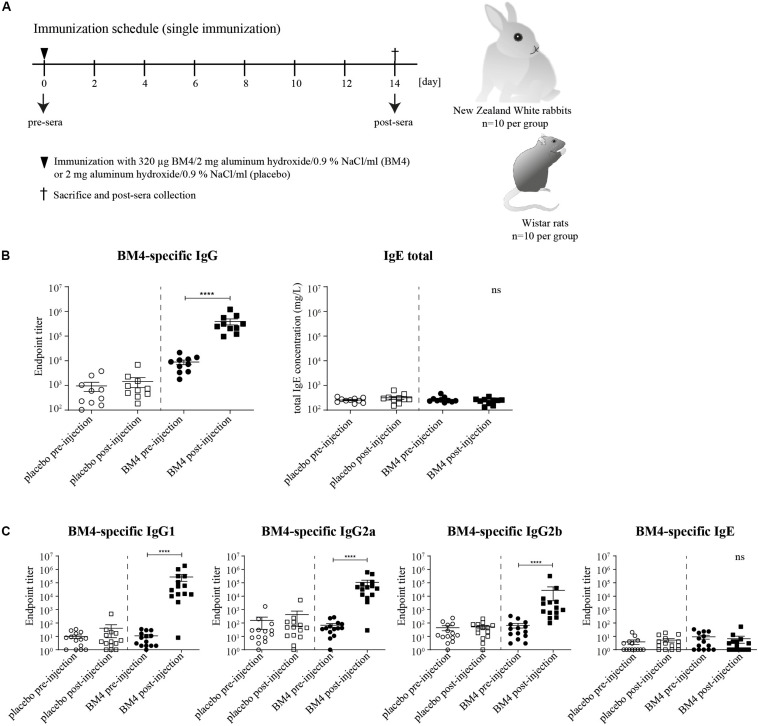
Immunization schedule of the single immunization model **(A)**. Wistar rats or NZW rabbits (each *n* = 10 per group) were immunized subcutaneously either with 320 μg BM4 or a corresponding adjuvant control (placebo). Rabbit BM4-specific IgG antibody titers and total IgE pre- and post-immunization were determined **(B)**. Rat BM4-specific IgG1, IgG2a, IgG2b, and IgE endpoint titers pre- and post-immunization **(C).** A paired *t*-test was performed to compare the pre- versus post-immunization values of each group.

**FIGURE 2:**
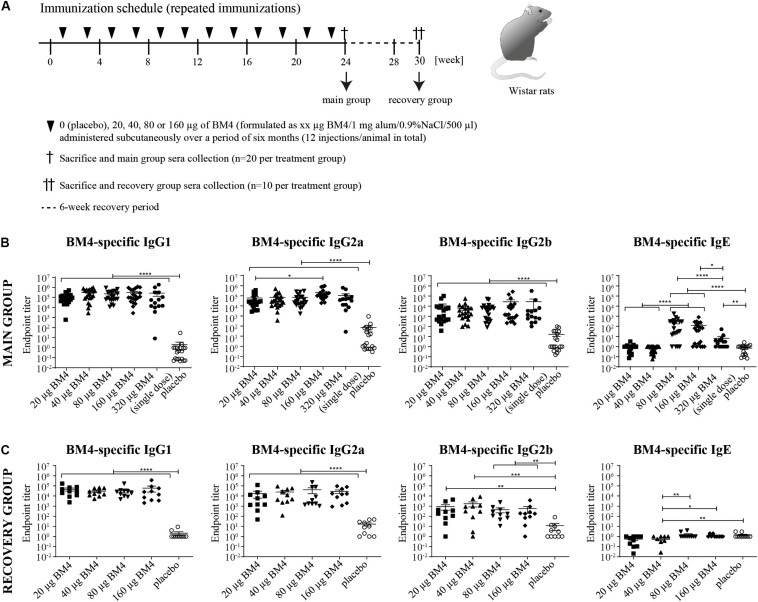
Immunization schedule of the repeated immunization model **(A)**. Wistar rats received bi-weekly s.c. immunizations with either 0 (placebo), 20, 40, 80, or 160 μg BM4 over a period of 6 weeks. Animals of the main group (*n* = 20 per treatment) were sacrificed 1 week after the last application, whereas animals of the recovery group (*n* = 10 per group) were sacrificed after a 6-week recovery period. Determination of BM4-specific IgG1, IgG2a, IgG2b, and IgE levels within rat sera of the main **(B)** and the recovery group **(C)** by ELISA. The single immunization of 320 μg BM4 (of the single immunization model) was only used for comparative purposes. The scatter plot depicts the mean of each treatment group and the SEM. Statistics were calculated on transformed data [Y = Log(Y)] using a one-way ANOVA. A Tuckey multiple comparison test was used to compare all groups with each other. ns >0.05; *p ≤ 0.05; **p ≤ 0.01; ***p ≤ 0.001; ****p ≤ 0.0001.

**FIGURE 3:**
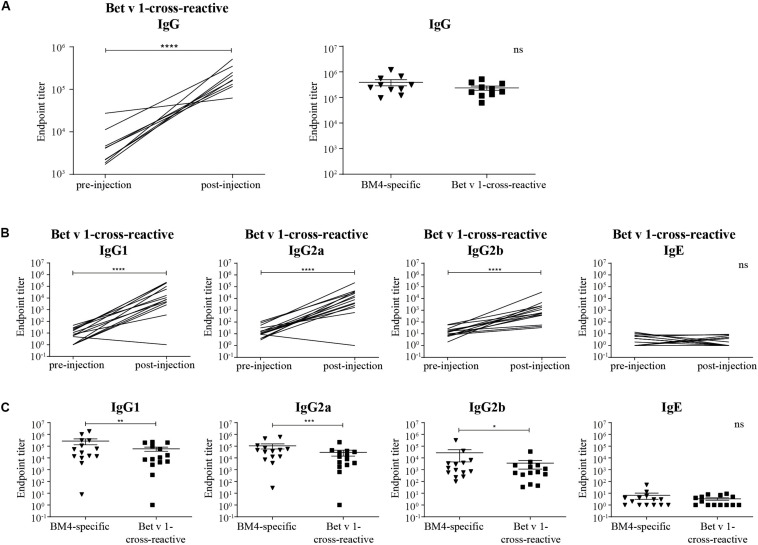
Rabbit Bet v 1-cross-reactive IgG antibody titers pre- and post-immunization (**A**, left) and direct comparison of rabbit BM4-specific and Bet v 1-cross-reactive IgG titers (**A**, right). Rat Bet v 1-cross-reactive IgG1, IgG2a, IgG2b, and IgE endpoint titers pre- and post-immunization **(B)** and pairwise comparison of BM4-specific and Bet v 1-cross-reactive IgG1, IgG2a, IgG2b, and IgE levels **(C)**. For statistical analysis, a paired *t*-test was performed. ns >0.05; *p ≤ 0.05; **p ≤ 0.01; ***p ≤ 0.001; ****p ≤ 0.0001.

**FIGURE 4:**
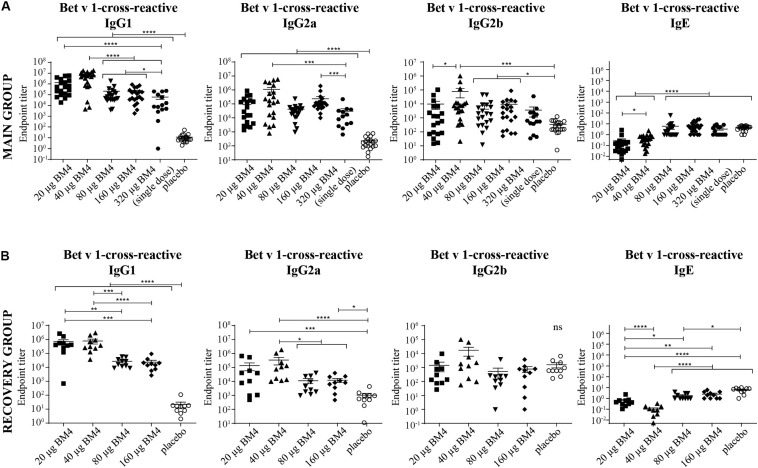
Combined data of Bet v 1-cross-reactive IgG1, IgG2a, IgG2b, and IgE titers of rat sera of the main **(A)** and the recovery group **(B)** of the repeated immunization model evaluated by ELISA. The mean and SEM is shown in each scatter plot. Statistics were calculated on transformed data [Y = Log(Y)] using a one-way ANOVA and a Tuckey multiple comparison test. The single immunization of 320 μg BM4 (of the single immunization model) was used for comparative reasons. ns >0.05; *p ≤ 0.05; **p ≤ 0.01; ***p ≤ 0.001; ****p ≤ 0.0001.

**FIGURE 5:**
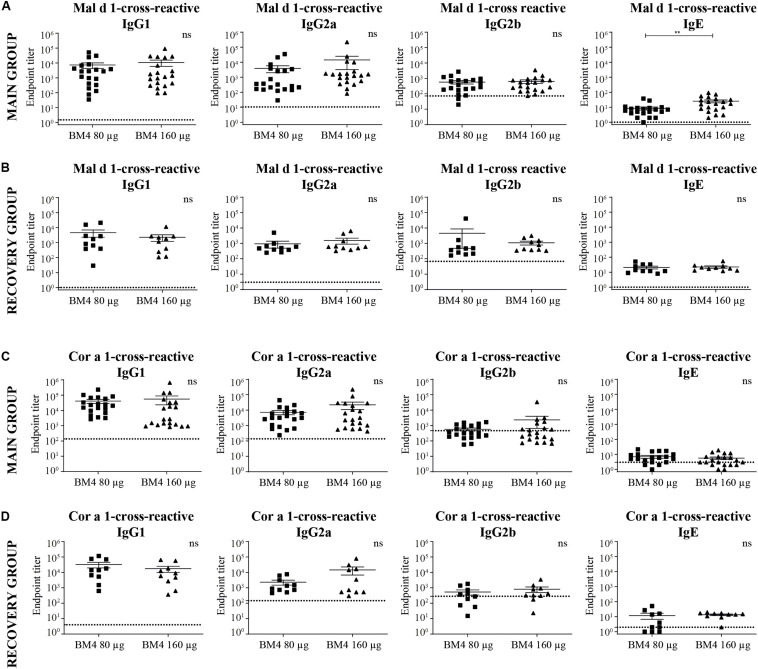
Analysis of Mal d 1- **(A,B)** and Cor a 1-cross-reactive **(C,D)** IgG1, IgG2a, IgG2b, and IgE titers of sera derived from Wistar rats receiving repeated immunization of either 80 or 160 μg BM4 (**A,C**: main group; **B,D**: recovery group). The dotted line represents a sera pool of animals receiving placebo. For statistical analysis, an unpaired *t*-test was performed on transformed [Y = log(Y)] data. ns >0.05; **p ≤ 0.01.

**FIGURE 6:**
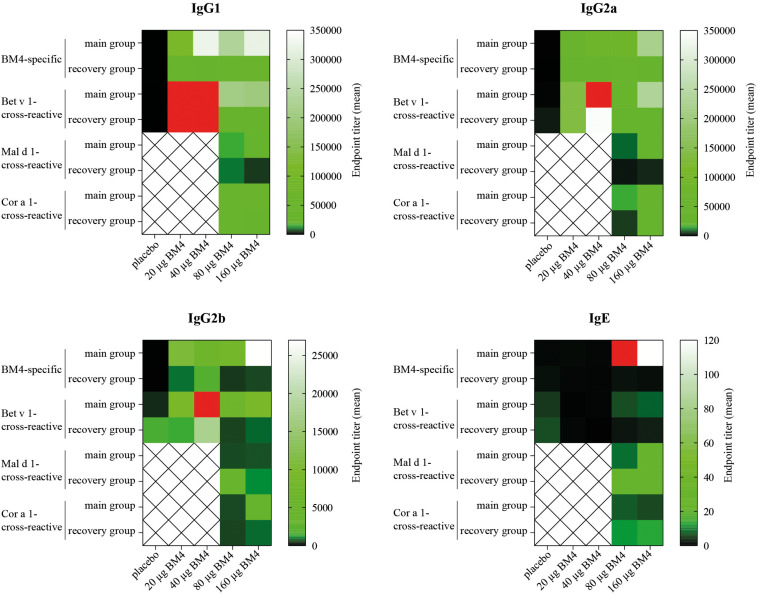
Comparative heat map showing the mean BM4-specific, and Bet v 1-, Mal d 1-, and Cor a 1-cross-reactive titers of each treatment group (either 0, 20, 40, 80, or 160 μg BM4) of Wistar rat sera of the repeated immunization model for each IgG subclass as well as for IgE. Red squares were used to highlight values that exceeded the titer range shown in the heat map.

**FIGURE 7:**
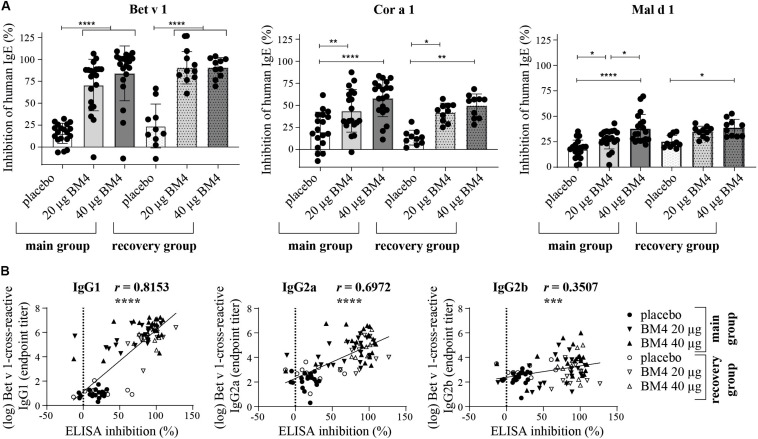
Inhibition of human IgE binding to Bet v 1, Cor a 1, and Mal d 1 by sera derived from Wistar rats receiving repeated immunizations of either 0 (placebo), 20, or 40 μg BM4 **(A)**. The column bar charts depict the mean and standard deviation of each data set. Statistics were calculated using a one-way ANOVA and a Dunnett’s multiple comparisons test comparing the individual main or the recovery groups among each other. Correlation of log-transformed Bet v 1 cross-reactive IgG1, IgG2a, and IgG2b titers with inhibition ELISA data using the Pearson correlation test **(B)**. ns >0.05; *p ≤ 0.05; **p ≤ 0.01; ***p ≤ 0.001; ****p ≤ 0.0001.

Data in the text are presented as mean ± standard error of the mean (SEM). Statistics were calculated on transformed data [Y = Log(Y)]. A paired *t*-test was used to compare pre- and post-immunization sera (Figures 1, 3A–B) and BM4-specific with Bet v 1-specific titers (Figure 3C), whereas an unpaired t-test was performed for the comparison of Mal d 1- and Cor a 1-specific antibody titers of the 80 μg BM4 treatment group with the 160 μg group (Figure 5). For comparing more than two sample groups, a one-way ANOVA was performed (Figures 2, 4, 6, 7). For the correlation of the inhibition ELISA and inhibition FAB assay data with the transformed Wistar rat IgG titers a Pearson correlation test was performed. All statistical analyses were performed using the GraphPad Prism 8.0 software (GraphPad Software, San Diego, CA, United States). The *p-*values were reported in the following way; ns > 0.05; ^∗^*p* ≤ 0.05; ^∗∗^*p* ≤ 0.01; ^∗∗∗^*p* ≤ 0.001; ^****^p ≤ 0.0001.

## Results

### A Single Immunization With BM4 Induces High Levels of Antigen-Specific IgG Antibodies in Wistar Rats and NZW Rabbits

To investigate in course of an initial screening the ability of the hypoallergenic molecule to induce an efficient antigen-specific IgG immune response in two different mammalian species, we determined the IgG antibody titers 2 weeks post-immunization with 320 μg BM4 (Figure 1A). In NZW rabbits, BM4 induced a 44.5-fold increase (*p* < 0.0001) of the mean antigen-specific IgG level post-immunization compared to the levels of the placebo control that remained unaltered (Figure 1B, mean titer: 8,828.7 pre-immunization and 393,177.8 post-immunization). In Wistar rats, we determined the antigen-specific IgG1, IgG2a, and IgG2b antibody titers in the pre- and post-immunization sera and compared them to the placebo control receiving only the adjuvant without antigen (Figure 1C). In the BM4 treatment group, the titers of the rat IgG antibody subclasses were significantly elevated following injection with the hypoallergen (*p* < 0.0001). The mean titers increased 24,721-fold for IgG1, 1,560-fold for IgG2a, and 402-fold for IgG2b (mean post-immunization titers: 271,932, 107,199, and 27,264, respectively). In comparison, the IgG titers of the placebo group, pre- and post-immunization, showed no change. Additionally to the IgG antibody titers, we sought to monitor the antigen-specific IgE levels in order to evaluate possible IgE-mediated side effects caused by the treatment. The treatment did not affect the total IgE level within rabbit sera (Figure 1B). In Wistar rats, the treatment with BM4 did not induce BM4-specific IgE (Figure 1C).

### Elevated Specific IgG Titers Persist in the Sera of Wistar Rats After Discontinuation of Repeated Immunizations With BM4

In order to mimic a standard AIT protocol using BM4 and to evaluate its consequences on the humoral immune response, we immunized naïve Wistar rats with BM4 bi-weekly over a period of 6 months (Figure 2A, main group). To assess the levels of IgG antibodies after treatment discontinuation, we analyzed the BM4-induced IgG antibody titers 6 weeks after the last injection (recovery group). The animals were immunized with either 0 (placebo), 20, 40, 80, or 160 μg BM4. In contrast to the placebo group, all individual animals of the main group receiving the hypoallergen showed a dose-independent increase of IgG titers comparable to the single application of 320 μg presented in Figure 1C (Figure 2B, *p* < 0.0001). In contrast to the single immunization model, the highest doses of BM4 (80 and 160 μg) in the repeated immunization model also caused a slight elevation of BM4-specific IgE antibodies compared to the placebo group (mean IgE titer: 389 and 169, respectively, *p* < 0.0001). On average, IgG1 and IgG2a levels were still 500 to 1,000-fold higher than IgE. Even the IgG2b titers, which increased less compared to IgG1 and IgG2a, were still 63.4-fold higher than the IgE levels. Interestingly, it appears that there are two types of responses among animals, one with increased IgE and the other with no alterations in the IgE levels.

Regarding the recovery group, the mean of BM4-specific IgE levels of each treatment group dropped below 1.5 resembling the placebo group. Of note, the 40 μg BM4 group was even significantly lower than the placebo group (*p* = 0.0069, Figure 2C). By contrast, all IgG levels of each BM4 treatment group remained significantly increased compared to placebo. The mean IgG1 titers ranged from 29,713 for 40 μg up to 59,722 for 160 μg BM4, and exceeded the placebo titer (2.1) by 14,149-fold to 28,439-fold (*p* < 0.0001). The determined mean IgG2a antibody titers were 17,969, 24,764, 40,608, and 28,456 for 20, 40, 80, and 160 μg BM4, respectively, and, thus, exceeded the placebo group (16.8) on average 1,663-fold. The IgG2b levels induced by the treatment with BM4 were approximately 30-times lower than the corresponding IgG1 and IgG2a titers. The highest antigen-specific mean IgG2b titer compared to placebo (11.6) was observed for the 40 μg BM4 (1,725, *p* = 0.0006) treatment group, followed by 20 μg (879, *p* = 0.0022), 160 μg (559, *p* = 0.0064), and 80 μg (446, *p* = 0.0019). A statistical comparison of the main and the recovery group of BM4-specific antibody titers, as well as of the following Bet v 1-, Mal d 1-, and Cor a 1-cross-reactive antibodies can be found in Supplementary Table S1.

### IgG Antibodies Induced by Immunization With BM4 Are Cross-Reactive With Wild-Type Bet v 1

For a potential application of the hypoallergen as an AIT vaccine in humans a functional confirmation of BM4-induced IgG antibodies recognizing the wild-type allergen is mandatory. In this respect, we first determined the Bet v 1-specific IgG antibody titers of sera derived from the single immunization (Figure 1A) as well as the repeated immunization model (Figure 2A). The IgG antibodies induced by a single immunization with BM4 were cross-reactive with Bet v 1 in both mammalian species (Figures 3A,B). The induction of Bet v 1-cross-reactive antibodies in the post-sera was significantly different for NZW rabbit IgG and Wistar rat IgG1, IgG2a, and IgG2b compared to the sera before immunization (all *p* < 0.0001). Side-by-side comparison of BM4-specific and Bet v 1-cross-reactive titers of the post-immunization sera revealed that the Bet v 1-cross-reactive IgG1, IgG2a, and IgG2b levels were marginally but significantly lower than the corresponding BM4-specific values (Figure 3C, *p* = 0.0022, *p* = 0.0003, and *p* = 0.0101, respectively). In contrast, no difference was observed in NZW rabbit post-sera (Figure 3A). In Wistar rats, a more ambiguous pattern was found for the Bet v 1-specific titers. Again, the Bet v 1-cross-reactive antibody titers for all three IgG subclasses of the main group were significantly increased compared to placebo (Figures 4A, 6), but in contrast to the single immunization model, the Bet v 1-specific IgG1 titers of the repeated immunization model, using 20 (*p* = 0.0011), 40 (*p* < 0.0001), and 160 μg BM4 (*p* = 0.0271), were actually significantly higher than the corresponding BM4-specific titers. In all three investigated IgG subclasses the repeated immunizations with 40 μg BM4 resulted in the highest mean Bet v 1-cross-reactive titers (IgG1: 5937,601.5, IgG2a: 1104,869.1, and IgG2b: 78,436.6), followed by 20 μg BM4 (IgG1: 1355,412.6, IgG2a: 139,285.0, and IgG2b: 9,798.1). The mean titers induced by 80 and 160 μg BM4 were comparatively lower. A similar pattern was observed for IgG1 and IgG2a titers of the recovery group (Figures 4B, 6). In contrast to the main group, Bet v 1-cross-reactive IgG2b titers of animals receiving repeated immunizations with BM4 did not differ from the placebo group. Compared to the induction of slight BM4-specific IgE levels, neither in the single immunization model (Figure 3B) nor the repeated immunization model (Figures 4A,B) elevated Bet v 1-cross-reactive IgE titers were observed.

### Wistar Rat IgG Antibodies Induced by Repeated Immunization With BM4 Are Cross-Reactive With the Bet v 1-Homologous Food Allergens Mal d 1 (Apple) and Cor a 1 (Hazelnut)

To investigate if treatment-induced antibodies bind to other allergens of the PR-10 family, we determined the titers of Mal d 1- and Cor a 1-cross-reactive IgG and IgE in rat sera derived from the repeated immunization model receiving the two highest BM4 dosages (80 and 160 μg). These food allergens share a sequence similarity of 56.6% (Mal d 1) and 67.3% (Cor a 1) with Bet v 1 and exhibit the common PR-10 fold. The structural integrity and similarity of the recombinant proteins used in this study with wild-type Bet v 1 was assessed by determining the secondary structural elements of each protein by CD and FTIR (Supplementary Figure S2). A high structural similarity between Bet v 1 and its homologous food allergens was observed. In contrast, BM4 exhibited a rather unfolded state using CD, whereas some structural features of the hypoallergenic molecule were still detectable when using FTIR for the analysis.

In direct comparison with the induced mean Bet v 1-specific IgG titers, it was apparent that all investigated Cor a 1- as well as Mal d 1-specific IgG subclasses were markedly lower (Figures 5A,C, 6 and Supplementary Table S2). Depending on the BM4 immunization dose, Mal d 1-specific IgG1 was either 18.6- to 28.9-fold lower than its Bet v 1-specific counterpart, and Mal d 1-specific IgG2a and IgG2b titers were 10.8- to 16.3-fold reduced. Notably, the induced Cor a 1-specific IgG titers were higher compared to their Mal d 1-specific corresponding values. Cor a 1-specific IgG1 was only 3.6- to 5.1-fold lower than the Bet v 1-specific IgG1 titers, IgG2a 5.8- to 10.5-fold, and IgG2b 4.1- to 12.9-fold. In the recovery group, except for Mal d 1-specific IgG2b, all Mal d 1-, and Cor a 1-specific mean titers of the investigated IgG subclasses dropped compared to the main group (Figures 5B,D, 6 and Supplementary Table S2). Still, compared to the Bet v 1-specific IgG titers of the recovery group, all mean titers of the Mal d 1- and Cor a 1-specific IgG subclasses were in a similar range; except for Mal d 1-specific IgG1 and IgG2a, which were 5.7- to 10.3- and 8.0- to 12.1-fold lower, respectively. Both, Mal d 1- and Cor a 1-specific IgE titers of the main and the recovery group were in a comparable range to Bet v 1-specific IgE, and thus hardly induced by the treatment with BM4.

Summarizing the specificity of the BM4-induced antibodies of the repeated immunization model, we can conclude that the highest IgG antibody levels were induced toward the wild-type Bet v 1 allergen rather than its hypoallergenic variant (Figure 6). IgG antibodies against the Bet v 1-homologous food allergens Cor a 1 and Mal d 1 were also induced but comparably at a lower extent. The highest IgE antibody levels were induced by 80 and 160 μg of BM4, and the specificity of these antibodies was also mostly directed toward BM4. However, in course of the toxicity study it became evident that the higher BM4 dosages (80 and 160 μg) exceeded the no-observed-adverse-effects level (NOAEL, data not shown), whereas the lower concentrations (20 and 40 μg) were considered “safe,” enabling the further investigation of the hypoallergen in a first-in-men human clinical trial. In this respect, only sera of rats immunized with 20 and 40 μg BM4 were subjected to further functional characterization.

### BM4-Induced Wistar Rat IgG Antibodies Inhibit Specific Binding of Human IgE to Bet v 1, Cor a 1, and Mal d 1

The induction of blocking antibodies (IgG4/IgG1) is a hallmark of successful AIT, thus, a sole characterization of the antibody pattern as well as of their specificity is not sufficient enough to draw conclusions of the functional relevance of the induced antibodies regarding the IgE-blocking activity of IgG antibodies. Therefore, we decided to investigate to which extent rat sera of the repeated immunization model (20 and 40 μg BM4) containing BM4-induced antibodies are able to compete with human IgE antibodies for binding of wild-type Bet v 1, Mal d 1, and Cor a 1. For this purpose, we used an inhibition ELISA (Figure 7A and Supplementary Table S3). In rat sera of the main group, immunization with BM4 (20 and 40 μg) induced a pronounced mean inhibition of human IgE binding to Bet v 1 (70.8 and 84.1%), a moderate mean inhibition to Cor a 1 (43.9 and 58%), and a low mean inhibition to Mal d 1 (27.6 and 37.6%). Whereas, placebo induced a mean inhibition of 15.6, 20.1 and 17.6% to Bet v 1, Cor a 1, and Mal d 1, respectively. Overall, the inhibitory capacity remained in sera of the recovery group (Bet v 1: 90.5%; Cor a 1: 42 and 49.5%; Mal d 1: 34.7 and 38.6%). However, for Mal d 1, immunization of 20 μg BM4 were not significantly different compared to placebo. For the placebo recovery group, an inhibition of 24% (Bet v 1), 13.4% (Cor a 1), and 25% (Mal d 1) was observed.

In summary, a mean difference of 61.8% (Bet v 1), 30.8% (Cor a 1), 15% (Mal d 1), and 66.4% (Bet v 1), 32.3% (Cor a 1), 11.65% (Mal d 1) was observable in the main and recovery group, respectively, compared to placebo. For Bet v 1, the inhibition results positively correlated with the respective cross-reactive IgG1 and IgG2a titers (*r* = 0.8153, *p* < 0.0001, and *r* = 0.6972, *p* < 0.0001, respectively), whereas the IgG2b titers showed a relatively low correlation with the inhibition of human IgE binding (*r* = 0.3507, *p* < 0.0008, Figure 7B).

### BM4-Induced Wistar Rat IgG Antibodies Are Able to Effectively Inhibit CD23-Mediated Human IgE-Facilitated Bet v 1 Binding

**FIGURE 8:**
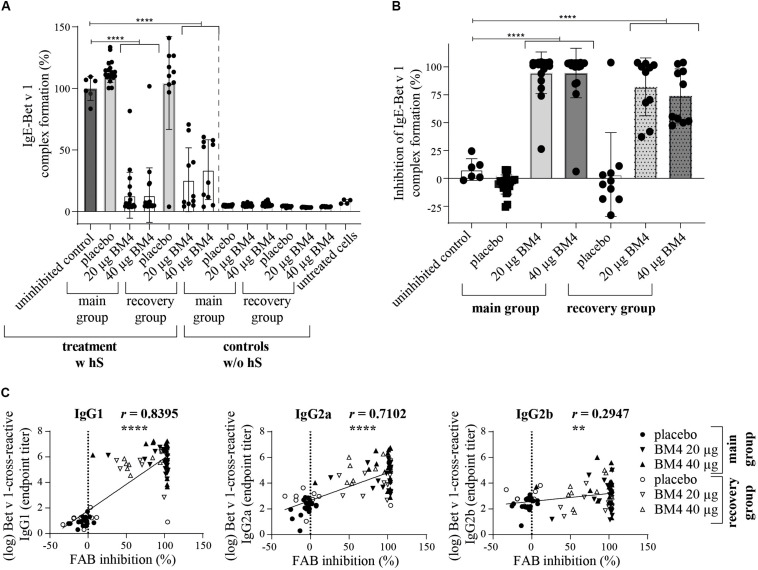
BM4-induced rat IgG antibodies are able to sufficiently inhibit CD23-mediated complex formation of Bet v 1 and human IgE antibodies on B cells. The inhibition FAB assay was performed with sera of rats of the repeated immunization model receiving either 0 (placebo), 20, or 40 μg BM4. The percentage of IgE-Bet v 1 complex formation **(A)** was normalized toward the mean of the uninhibited control (100%). Untreated cells were used as negative controls. For the evaluation of unspecific rat IgE-Bet v 1 complex formation, controls without human serum (w/o hS) were included; w hS, with human serum. The percentage inhibitory activity was calculated by subtracting the baseline activation of untreated cells followed by subtraction of the normalized IgE-Bet v 1 complex formation values from 100 **(B)**. The column bar charts depict the mean and standard deviation of each data set. Statistics were calculated using a one-way ANOVA and a Dunnett’s multiple comparisons test comparing each treatment group with the uninhibited reference. Statistics of the controls (w/o hS) were excluded due to simplicity reasons but significantly lower than the uninhibited control (positive control, *p* = 0.0001). A Pearson correlation test was performed to analyze if the determined Bet v 1-cross-reactive IgG1, IgG2a, and IgG2b titers were correlating with the inhibition of IgE-Bet v 1 complex formation **(C)**. ns >0.05; **p ≤ 0.01; ****p ≤ 0.0001.

In addition to the inhibition ELISA data, we sought to investigate whether the BM4-induced antibodies are able to interfere with the CD23-mediated binding of Bet v 1 facilitated by human IgE. Therefore, the inhibition facilitated antigen-binding (inhibition FAB) assay protocol by Shamji et al. (26) was adapted accordingly. Sera of Wistar rats receiving repeated immunizations with BM4 (20 and 40 μg) caused a significant reduction of Bet v 1-IgE complexes binding to B cells (*p* < 0.0001) compared to placebo in the main as well as in the recovery group (Figure 8A). A mean decrease of 86% of Bet v 1-IgE complexes was observed for sera of the main group, whereas for the recovery group a treatment with both dosages of BM4 resulted in a lowered reduction of 66% (40 μg) and 74% (20 μg). Although the IgE of rat sera were heat-inactivated in order to solely assess the capability of human IgE antibodies to provoke allergen-IgE complex formation, we screened the rat sera toward their insufficiency to cause the formation of complexes in the absence of human IgE (controls without human serum). None of the heat-inactivated rat sera was *per se* able to form allergen-IgE complexes. To determine the percentage of inhibitory activity of the BM4-induced IgG antibodies we converted the experimental values after baseline subtraction (untreated cells) by inverting the percentage of allergen-IgE complexes (mean of uninhibited controls minus the IgE-Bet v 1 complex formation values). An efficient mean inhibitory activity of 95% was found for sera of rats receiving BM4 of the main group (*p* < 0.0001), and 74% (40 μg, *p* < 0.0001) to 82% (20 μg, *p* < 0.0001) for those of the recovery group (Figure 8B). A positive correlation was observed between the FAB inhibition results and the Bet v 1-cross-reactive IgG1 and IgG2a titers (*r* = 0.8395, *p* < 0.0001, and *r* = 0.7102, *p* < 0.0001, respectively), whereas there was a relatively low correlation with the Bet v 1-cross-reactive IgG2b titers (*r* = 0.2947, *p* < 0.0053, Figure 8C).

## Discussion

Allergen-specific immunotherapy is the only curative approach modulating the causative cellular and molecular origin of allergic diseases by skewing the Th2-biased IgE-mediated inflammatory response toward an anti-inflammatory immune response resulting eventually in allergen immunotolerance (22, 23). The underlying protective mechanisms involve the induction of antigen-specific regulatory T and B cells, and the secretion of immunosuppressive cytokines, such as IL-10 (23, 33, 34). Another key regulatory molecular event in the induction of immunotolerance by AIT and, consequently, the reduction of allergic symptoms, is the increased production of allergen-specific IgG4 antibodies. These antibodies are able to prevent antigen recognition by IgE and the consecutively occurring effector functions, including degranulation of mast cells and basophils, histamine release, and the recruitment of various inflammatory cells (25, 35). This allergen-neutralizing functional aspect of serum IgG4 has been reported as a sufficient predictive biomarker for AIT efficacy (25, 26, 32). Following this idea, we aimed to monitor the IgG antibody levels induced by a hypoallergenic variant of Bet v 1 in two different naïve mammalian animal models (i), and to characterize their specificity toward wild-type Bet v 1 (ii) as well as to the Bet v 1-associated food allergens Mal d 1 and Cor a 1 (iii). We further analyzed the functionality of the IgG antibodies regarding their activity to prevent human IgE-allergen binding (iv). We found that the treatment with BM4 induced elevated Bet v 1-specific IgG antibody titers in both mammalian species. In Wistar rats, high BM4- and Bet v 1-specific IgG1, IgG2a and IgG2b levels (10^4^ to 10^6^) were induced even upon receiving just a single immunization. Since neither Wistar rats nor NZW rabbits possess the IgG4 subclass, we sought to cover the complete IgG repertoire among these species, except for rat IgG2c that is primarily recognizing carbohydrate epitopes (36–38). While rats have four IgG subclasses (IgG1, IgG2a, IgG2b, and IgG2c), NZW rabbits only possess a single IgG subclass (29). Although an interspecies comparison of IgG subclasses is difficult, the general consensus is that rat IgG1 and IgG2a functionally resemble human IgG4/murine IgG1. Conversely, rat IgG2b corresponds to human IgG1/murine IgG2a/2b (36–41).

In mice, immunizations with BM4 resulted in a boosted Bet v 1-specific IgG1 and IgG2a antibody secretion and were associated with a Th1-skewing effect (18). Immunizations with Bet v 1, on the other hand, hardly induced an upregulated secretion of these antibodies, which is in line with our findings of the repeated immunization model showing increased Bet v 1-specific titers compared to BM4-specific antibodies. Since the structure of BM4 differs fundamentally from wild-type Bet v 1, we hypothesize that this treatment-induced antibody cross-reactivity mainly occurs due to the recognition of sequential epitopes by the IgG antibodies. However, in contrast to our findings, where BM4 did hardly affect Bet v 1-specific IgE antibody levels, BM4 also resulted in a quick elevation of Bet v 1-specific IgE in a Balb/c immunization model (18). Especially in the case of Bet v 1, the dominant IgE epitopes mostly possess a conformational nature, which would explain why elevated BM4-specific IgE titers derived from our repeated immunization (80 and 160 μg BM4) hardly recognized Bet v 1, Mal d 1, and Cor a 1 (42). In the recovery group, no BM4-specific IgE was detectable, most likely due to the short-lived nature of IgE. Due to the non-existence of IgE memory cells, the production of IgE antibodies depends on a constantly active Th2-biased inflammatory milieu promoting the class switching of B cells to IgE-secretory plasma cells (43). In immunization models such an inflammatory milieu is provided by the co-administration of an adjuvant. Therefore, treatment discontinuation resulted in a drop of IgE titers in the recovery group. The reduced reactivity of Mal d 1 and Cor a 1 by BM4-induced antibodies compared to Bet v 1 most likely results from the differences in sequence identity between the recombinant wild-type and the mutant proteins (BM4:Bet v 1 96.86%; BM4:Mal d 1 59.75%; BM4:Cor a 1 64.78%). This explains why Mal d 1 is even less recognized than Cor a 1 in both, the main as well as the recovery group of the repeated immunization model, and why human IgE binding to Mal d 1 is also less efficiently inhibited.

By using the inhibition ELISA and inhibition FAB assay, we were able to address the functional relevance of the BM4-induced antibodies and found that sera of BM4-imunized rats were able to significantly neutralize the binding of Bet v 1 to human IgE. The inhibitory activity induced by BM4 even remained after treatment discontinuation. This and the fact that the results obtained for both assays positively correlated with the Bet v 1-cross-reactive IgG1 and IgG2a titers corroborates to our assumption that this blocking activity occurs due to the increased Bet v 1-specific IgG levels. However, further experiments are needed in order to clearly state, which rat IgG subclass is responsible for the IgE blocking activity. Also, additional *in vitro* approaches addressing the functionality of the induced IgG antibodies in other functions of effector cells, such as the basophil activation test or mediator release assays, would provide further insights on the inhibitory capacity of the BM4-induced IgG antibodies (27, 44). In a therapeutic *in vivo* model, increased Bet v 1-specific IgG1 levels induced by intraperitoneal injections of BM4 were associated with a downregulation in Bet v 1-triggered mediator release using rat basophilic leukemia cells, as well as a general decrease of Th2-mediated inflammation as judged by BALF IL5 cytokine secretion and lung infiltrating cells (20).

In general, treatment with recombinant Bet v 1 was proven successful providing a potential alternative to extract-based AIT vaccines (45, 46). By providing an efficient hypoallergen-based drug product to the market that, ideally, tackles the negative aspects of AIT (long treatment duration and treatment-induced side effects), would certainly increase the popularity of AIT and demonstrate as well as magnify its to date unexploited potential. Other hypoallergenic Bet v 1 derivatives were lacking statistical difference regarding their efficacy compared to current birch pollen extract-based AIT protocols (47). Our data, showing that BM4 induces high and sustainable IgG levels in two different mammalian species, highlight the suitability of BM4 as a hypoallergenic drug candidate for birch pollen AIT, with the potential of reducing birch-associated PFAS symptoms.

## Data Availability Statement

The raw data supporting the conclusions of this article will be made available by the authors, without undue reservation.

## Ethics Statement

The animal study was reviewed and approved by the National Animal Experiment Board of Finland. Ethical approval for using human serum of allergic patients was obtained by the Dutch Ethical Committee (number: NL65758.018.18).

## Author Contributions

LA devised and performed most experiments, wrote the manuscript, and created the figures. AB and MG conducted the experiments. PC, U-MJ, and EY designed, organized, and performed the *in vivo* models. FS provided the formulated GMP drug products for the *in vivo* experiments and BM4 for the ELISAs. LJ and RV devised the experiments and interpreted the data. All authors read the manuscript. All authors contributed to the article and approved the submitted version.

## Conflict of Interest

FS is an employee of Biomay AG. The remaining authors declare that the research was conducted in the absence of any commercial or financial relationships that could be construed as a potential conflict of interest.

## Supplementary Material

FIGURE S1 SDS-PAGE gel of the recombinantly produced proteins used in this study. M, Pierce Unstained Protein Molecular Weight Marker (Thermo Fisher Scientific, Waltham, MA, United States).

FIGURE S2 CD spectra of BM4, Bet v 1, Mal d 1, and Cor a 1 recorded at 20°C between 190 and 260 nm **(A)**. Amide I and II and second derivative of amide I IR-spectra of the recombinant proteins recorded at 25°C **(B)**. The percentage of α-helical and β-strand-like content of secondary structural elements was calculated from the IR-spectra, and a comparison of the experimentally collected data for Bet v 1 and Mal d 1 with the respective theoretical values deposited on PDB is shown (code: 4A88 and 5MMU, respectively).

FIGURE S3 Definition of experimental conditions for the FAB assay. A titration of Bet v 1 concentration **(A)**, amount of human reference serum and anti-IgE antibody **(B)** was performed. Black arrows indicate the conditions used for the final inhibition FAB assay; w hS, with human serum (10 μl).

FIGURE S4 Inhibition FAB assay gating strategy for the analysis of Bet v 1-IgE complex immobilized on CD23-expressing EBV-transformed B cells. Cells were gated based on scatter light (FSC, SSC) characteristics, followed by doublet discrimination (SSC area versus SSC height). Living B cells were gated and analyzed toward anti-IgE binding (FITC, histogram, **A**) and/or CD23 (PE) expression (dot blot, **B,C**). Only the anti-IgE^+^ gated cells were considered for the analysis in Figure 7. The same gates were used for each sample. Results for positive control (uninhibited reference) and untreated cells (negative control) are shown.

## Abbreviations

AIT: allergen-specific immunotherapy
BSA: bovine serum albumin
CD: circular dichroism
ELISA: enzyme-linked immunosorbent assay
EBV: Epstein–Barr virus
FSC: forward scatter
FTIR: Fourier transform infrared
HRP: horseradish peroxidase
hS: human serum
inhibition FAB: inhibition IgE-facilitated allergen binding
L/D: live/dead
LOQ: limit of quantification
NZW rabbits: New Zealand White rabbits
PR-10: pathogenesis-related protein-10
PFAS: pollen food allergy syndrome
SCIT: subcutaneous immunotherapy
SSC: side scatter
SFP: specific pathogen-free
SEM: standard error of the mean
s.c.: subcutaneous.
