# Supplementary material for: In vivo Induction of Functional Inhibitory IgG Antibodies by a Hypoallergenic Bet v 1 Variant
Source: Front Immunol. 2020 Sep 3;11:2118. doi: 10.3389/fimmu.2020.02118 (PMC7494741; doi:10.3389/fimmu.2020.02118)
Supplement: Supplementary file 1 [file Table_1.DOCX]

Table S1. Statistical analysis of either BM4-specifc or Bet v 1-, Mal d 1- and Cor a 1-cross-reactive IgG1, IgG2a, IgG2b and IgE endpoint titers comparing the main group versus (vs.) the recovery group

| **Antigen specificity** | **Immunoglobulin class** | **BM4 immunization dosage (µg)** | ***p* value summary** | ***p* value** |
| --- | --- | --- | --- | --- |
| **BM4** | **IgG1** | 20 | ns | 0.1071 |
|  |  | 40 | ** | 0.0015 |
|  |  | 80 | *** | 0.0006 |
|  |  | 160 | ** | 0.0046 |
|  |  | placebo | * | 0.018 |
|  | **IgG2a** | 20 | ** | 0.0059 |
|  |  | 40 | ns | 0.0965 |
|  |  | 80 | * | 0.0207 |
|  |  | 160 | **** | <0.0001 |
|  |  | placebo | ns | 0.8974 |
|  | **IgG2b** | 20 | * | 0.0122 |
|  |  | 40 | * | 0.0199 |
|  |  | 80 | *** | 0.0005 |
|  |  | 160 | *** | 0.0008 |
|  |  | placebo | ns | 0.8735 |
|  | **IgE** | 20 | ns | 0.2167 |
|  |  | 40 | ns | 0.1324 |
|  |  | 80 | **** | <0.0001 |
|  |  | 160 | *** | 0.0008 |
|  |  | placebo | * | 0.0219 |
| **Bet v 1** | **IgG1** | 20 | ns | 0.4874 |
|  |  | 40 | ns | 0.0918 |
|  |  | 80 | * | 0.0193 |
|  |  | 160 | ** | 0.0096 |
|  |  | placebo | ns | 0.2505 |
|  | **IgG2a** | 20 | ns | 0.4722 |
|  |  | 40 | ns | 0.6884 |
|  |  | 80 | ** | 0.0063 |
|  |  | 160 | **** | <0.0001 |
|  |  | placebo | * | 0.0164 |
|  | **IgG2b** | 20 | ns | 0.4335 |
|  |  | 40 | ns | 0.2447 |
|  |  | 80 | ** | 0.0032 |
|  |  | 160 | * | 0.0159 |
|  |  | placebo | ** | 0.0054 |
|  | **IgE** | 20 | ** | 0.0036 |
|  |  | 40 | ** | 0.0031 |
|  |  | 80 | ns | 0.1797 |
|  |  | 160 | * | 0.0341 |
|  |  | placebo | ns | 0.3099 |
| **Mal d 1** | **IgG1** | 80 | ns | 0.7283 |
|  |  | 160 | ns | 0.3777 |
|  | **IgG2a** | 80 | ns | 0.82 |
|  |  | 160 | ns | 0.3354 |
|  | **IgG2b** | 80 | ns | 0.2561 |
|  |  | 160 | ns | 0.0769 |
|  | **IgE** | 80 | ** | 0.0017 |
|  |  | 160 | ns | 0.4948 |
| **Cor a 1** | **IgG1** | 80 | ns | 0.357 |
|  |  | 160 | ns | 0.9327 |
|  | **IgG2a** | 80 | ns | 0.1063 |
|  |  | 160 | ns | 0.6118 |
|  | **IgG2b** | 80 | ns | 0.3743 |
|  |  | 160 | ns | 0.9462 |
|  | **IgE** | 80 | ns | 0.5952 |
|  |  | 160 | ** | 0.0015 |

Table S2. Fold decrease of mean Mal d 1- and Cor a 1-cross-reactive antibody titers noticeably reduced compared to mean Bet v 1-cross-reactive antibody titers

| **Antigen**  **specificity** | **Immunization group** | **Immuno-globulin class** | **BM4 immunization dosage (µg)** | **-fold decrease compared to Bet v 1** | ***p* value** |
| --- | --- | --- | --- | --- | --- |
| **Bet v 1 vs. Mal d 1** | Main | IgG1 | 80 | 28.9 | <0.0001 |
|  |  |  | 160 | 18.6 | <0.0001 |
|  |  | IgG2a | 80 | 10.8 | <0.0001 |
|  |  |  | 160 | 16.3 | <0.0001 |
|  |  | IgG2b | 80 | 12.7 | 0.0299 |
|  |  |  | 160 | 14.9 | 0.1134 |
|  | Recovery | IgG1 | 80 | 5.7 | 0.0001 |
|  |  |  | 160 | 10.3 | 0.0109 |
|  |  | IgG2a | 80 | 12.1 | 0.0115 |
|  |  |  | 160 | 8.0 | 0.0292 |
| **Bet v 1 vs. Cor a 1** | Main | IgG1 | 80 | 5.1 | 0.0453 |
|  |  |  | 160 | 3.6 | 0.0001 |
|  |  | IgG2a | 80 | 5.8 | 0.0003 |
|  |  |  | 160 | 10.5 | 0.0003 |
|  |  | IgG2b | 80 | 12.9 | 0.0117 |
|  |  |  | 160 | 4.1 | 0.0259 |
|  |  |  |  |  |  |

Table S3. Additional significant statistics supplementing inhibition ELISA data

| **Antigen specificity** | **Group comparison** | ***p* value summary** | ***p* value** |
| --- | --- | --- | --- |
| **Bet v 1** | placebo vs. 20 µg BM4 recovery | **** | <0.0001 |
|  | placebo vs. 40 µg BM4 recovery | **** | <0.0001 |
|  | placebo recovery vs. 20 µg BM4 | **** | <0.0001 |
|  | placebo recovery vs. 40 µg BM4 | **** | <0.0001 |
| **Mal d 1** | placebo vs. 20 µg BM4 recovery | *** | 0.0004 |
|  | placebo vs. 40 µg BM4 recovery | **** | <0.0001 |
|  | placebo recovery vs. 40 µg BM4 | * | 0.0202 |
| **Cor a 1** | placebo vs. 40 µg BM4 recovery | ** | 0.0028 |
|  | placebo recovery vs. 20 µg BM4 | ** | 0.0018 |
|  | placebo recovery vs. 40 µg BM4 | **** | <0.0001 |
|  |  |  |  |
|  |  |  |  |
