# Supplementary figures and images for: In vivo Induction of Functional Inhibitory IgG Antibodies by a Hypoallergenic Bet v 1 Variant
Source: Front Immunol. 2020 Sep 3;11:2118. doi: 10.3389/fimmu.2020.02118 (PMC7494741; doi:10.3389/fimmu.2020.02118)

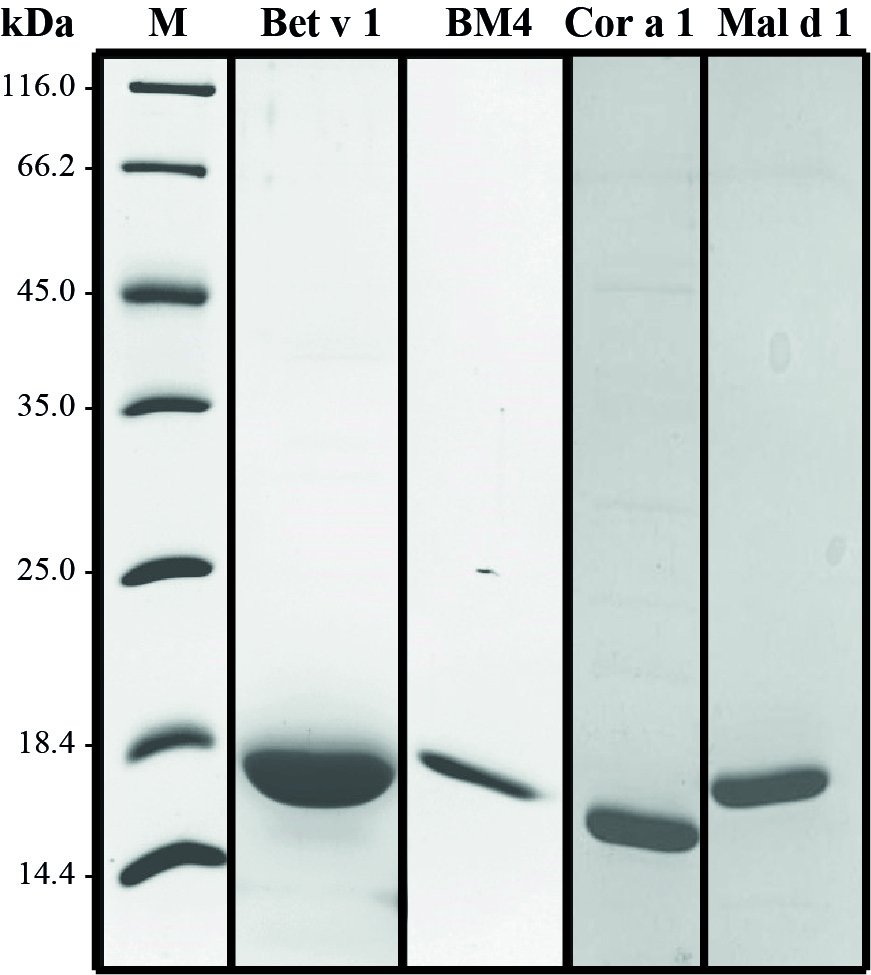

Supplement: FIGURE S1 — SDS-PAGE gel of the recombinantly produced proteins used in this study. M, Pierce Unstained Protein Molecular Weight Marker (Thermo Fisher Scientific, Waltham, MA, United States). [file Image_1.JPEG]

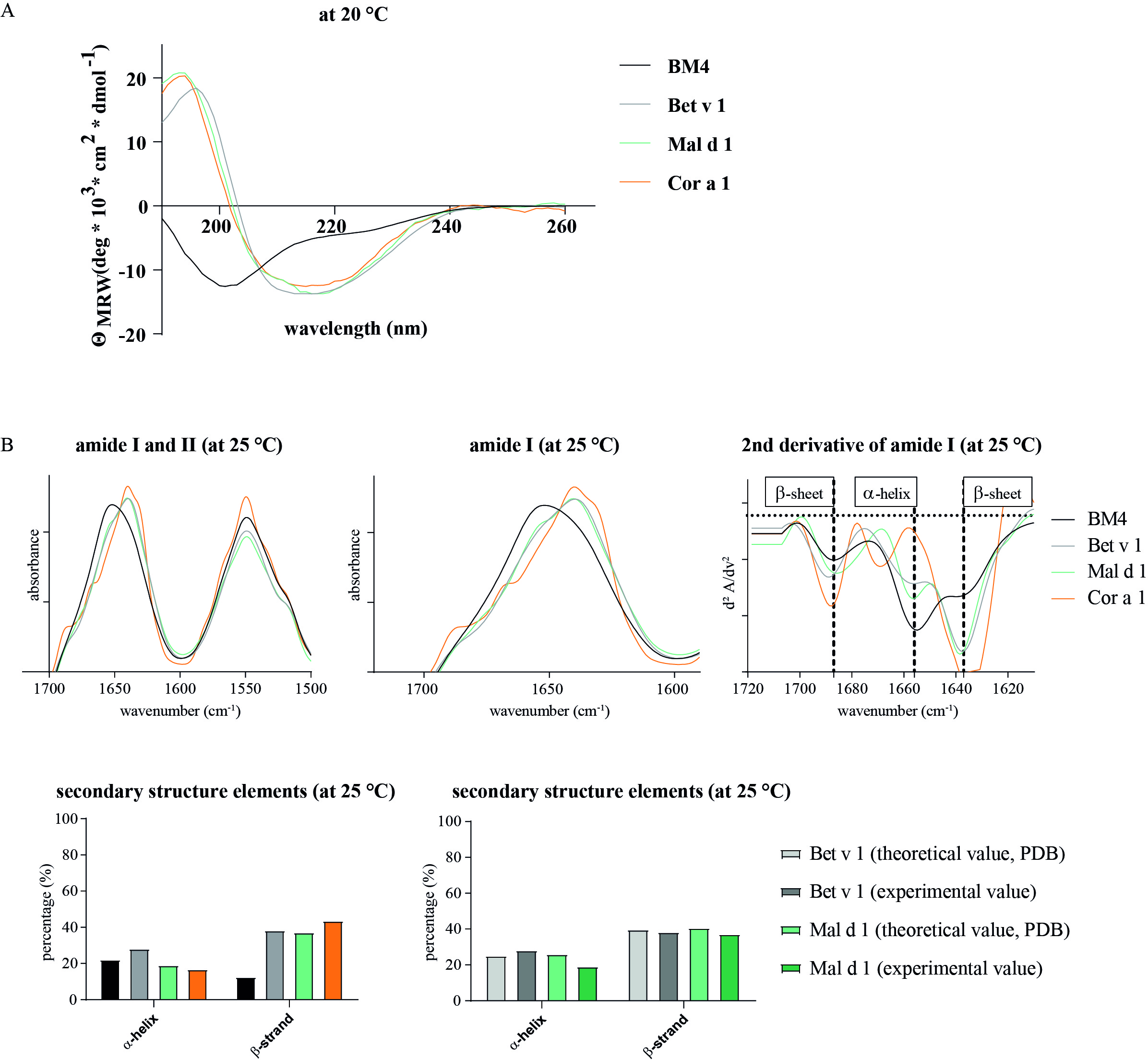

Supplement: FIGURE S2 — CD spectra of BM4, Bet v 1, Mal d 1, and Cor a 1 recorded at 20°C between 190 and 260 nm (A). Amide I and II and second derivative of amide I IR-spectra of the recombinant proteins recorded at 25°C (B). The percentage of α-helical and β-strand-like content of secondary structural elements was calculated from the IR-spectra, and a comparison of the experimentally collected data for Bet v 1 and Mal d 1 with the respective theoretical values deposited on PDB is shown (code: 4A88 and 5MMU, respectively). [file Image_2.JPEG]

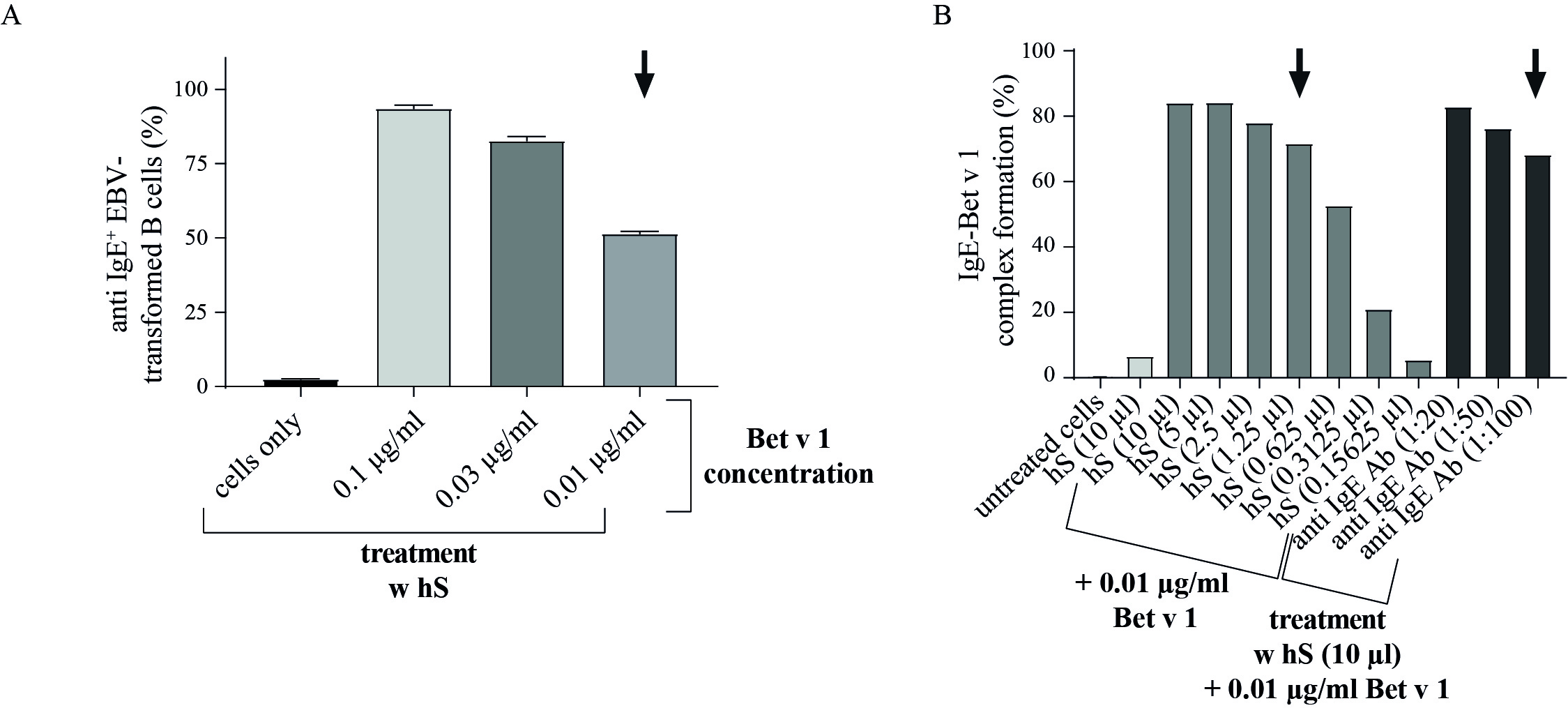

Supplement: FIGURE S3 — Definition of experimental conditions for the FAB assay. A titration of Bet v 1 concentration (A), amount of human reference serum and anti-IgE antibody (B) was performed. Black arrows indicate the conditions used for the final inhibition FAB assay; w hS, with human serum (10 μl). [file Image_3.JPEG]

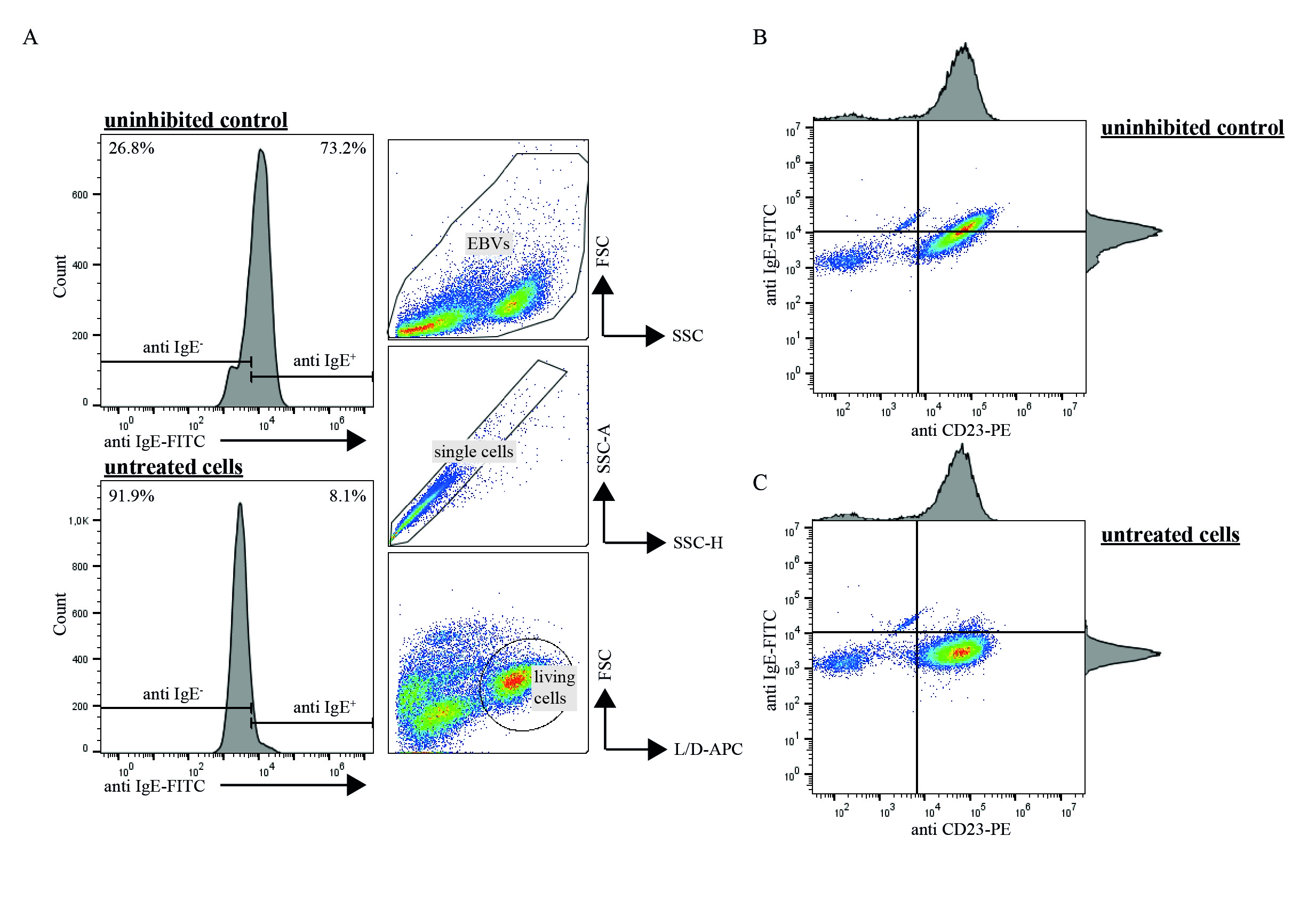

Supplement: FIGURE S4 — Inhibition FAB assay gating strategy for the analysis of Bet v 1-IgE complex immobilized on CD23-expressing EBV-transformed B cells. Cells were gated based on scatter light (FSC, SSC) characteristics, followed by doublet discrimination (SSC area versus SSC height). Living B cells were gated and analyzed toward anti-IgE binding (FITC, histogram, A) and/or CD23 (PE) expression (dot blot, B,C). Only the anti-IgE+ gated cells were considered for the analysis in Figure 7. The same gates were used for each sample. Results for positive control (uninhibited reference) and untreated cells (negative control) are shown. [file Image_4.JPEG]
